# Homonymous hemianopia in posterior cortical atrophy: right–left asymmetry, progression over time and relationship to the classical neuropsychological deficits

**DOI:** 10.3389/fneur.2026.1759440

**Published:** 2026-03-10

**Authors:** Mari-Nilva Maia da Silva, Merle James-Galton, Clare Green, Gordon T. Plant

**Affiliations:** 1 University College London Queen Square Institute of Neurology, London, United Kingdom; 2 Department of Neuropsychiatry, Hospital Nina Rodrigues, São Luís, Brazil; 3 The National Hospital for Neurology and Neurosurgery, London, United Kingdom; 4 King’s College London, London, United Kingdom

**Keywords:** Alzheimer disease, homonymous hemianopia, object perception, posterior cortical atrophy, Riddoch phenomenon, spatial perception

## Abstract

Following Benson’s seminal paper published in 1988 visual field loss in Posterior Cortical Atrophy (PCA) has been largely denied or ignored. This is despite an earlier description by Cogan of a similar case of pathologically verified Alzheimer disease featuring homonymous hemianopia (HH). Although HH is now recognised as a core feature of PCA its characteristics and relationship to other PCA features are unexplored. This study aimed to characterise the perimetric abnormalities in PCA patients presenting with HH, focusing on the response to static and kinetic stimuli; progression of the deficit over time; and the relationship to co-existing cognitive deficits. 24 patients were recruited for the cross-sectional study, of whom 19 participated in the longitudinal study. Each assessment consisted of kinetic and static perimetry and a comprehensive neuropsychological evaluation. The latter included tests to all basic cognitive domains plus tests of posterior cortical function. Thirteen patients underwent additional kinetic perimetry using three target velocities. Left HH was predominant and a deficit in object perception universal. Neglect was uncommon and did not correlate with the laterality of the HH. Stato-kinetic dissociation (SKD) was observed in all patients, greater in the more affected hemifield. Longitudinally, static perimetric deficits declined at a greater rate in the initially more affected hemifield. The rate of decline to kinetic testing was lower and varied with target size and velocity. Deterioration to the smallest and lowest velocity target mirrored that of static loss. A longitudinal mixed-model analysis showed that right HH was associated with greater deficits in predominantly left hemisphere cognitive functions and bilateral HH with greater bilateral occipital deficits. However, no association was found between the laterality of the HH and right hemisphere cognitive deficits. The characteristic SKD (also known as the Riddoch phenomenon) does not represent a complete dissociation as kinetic detection deteriorates over time in all cases. The correlation of the HH with lateralised parietal deficits challenges the concept that HH is restricted to an extreme posterior variant of PCA and highlights the need for routine (static and kinetic) perimetry in the diagnosis, characterisation and monitoring of PCA.

## Introduction

Posterior Cortical Atrophy (PCA) was syndromically defined in 1988 (1) as progressive visual and cognitive impairment characterised by elements of the syndromes of Bálint (simultanagnosia, ocular apraxia, optic ataxia) (2) and of Gerstmann (agraphia, acalculia, finger agnosia and right–left disorientation) (3). Other features were visual agnosia, alexia, and transcortical sensory aphasia without visual field loss. Parieto-occipital atrophy was identified radiologically in all cases, with involvement of temporo-occipital regions in some. The pathology was uncertain but Alzheimer disease suspected, thought at the time to spare primary sensory cortex (4). Three years prior to this seminal paper, Cogan reported a case manifesting as a visual cortical disorder, demonstrating visuospatial deficits, but also homonymous hemianopia (HH). Alzheimer pathology was confirmed at autopsy. Two additional patients had a similar history, no visual field defect, and clinical features suggestive of Alzheimer disease (AD) (5). A second autopsied case-study was later published as a “visual variant of AD” (6); the presence of visual field deficits and the similarity of the cases reported by Cogan and Benson was noted.

Notwithstanding these early observations, until recently, most accounts of the syndrome either excluded patients with visual field defects (7, 8), did not mention their presence or absence (9–11) or dismissed visual field loss as a consequence of neglect (12). Further reports, however, have since confirmed that HH in PCA patients is a common finding (13–20) and may indeed be present asymptomatically, before other symptoms(21–23). The phenotype of the visual deficit has, however, rarely been characterized (24). HH in PCA correlates with a greater degree of atrophy in the contralateral occipital lobe (25), confirming at least a fundamental relationship to the lateralization of the pathology. However, details of the pathological basis of HH in PCA; the characteristics of the residual vision; the relationship of visual field defects to the neuropsychological features; and their progression over time remain unexplored.

Sub-modalities of visual processing, such as motion detection, may be employed to more fully examine residual vision in PCA. The Riddoch phenomenon (relative preservation of motion detection) (26), for example, has been reported in a case series published by us (27) and in isolated case reports (22, 28). Progressive deterioration of cognitive functions is well established (29), but whether this is the case for early visual processing, such as threshold luminance increment detection that is the basis of perimetry, is unknown. The aim of the present study is to characterise the visual field abnormalities in a cohort of PCA patients presenting with HH, particularly in relation to the response to static and kinetic stimuli; progression of the deficit over time; and their relationship to co-existing cognitive deficits.

## Methods

### Patients

Patients were included in this study if they had: firstly a consensus classification (15) diagnosis of “PCA-pure” and secondly, homonymous visual field defects documented on the static Humphrey visual field test (SHVF). Seventeen patients were at level 2 of the consensus classification (without biomarker evidence of AD), the remainder at level 3 (with biomarker evidence of AD). The patients were selected from all PCA patients seen by GTP in a Neuro-Ophthalmology Clinic between 2010 and 2019 who either presented with or were found to have hemianopia on static perimetry. The other important criterion was the ability to attend for serial testing. Some of the patients were involved in neuro-imaging studies (25) but it was not possible to carry out a systematic or serial study in this cohort.

The total of twenty-four patients meeting these criteria consented to participate in this study, which was reviewed by the London–Queen Square Research Ethics Committee and approved under the number 16/LO/0264. All patients underwent a preliminary thorough clinical investigation, which included the following: detailed Neurological and Neuro-Ophthalmological examination, Neuropsychological assessment; screening for secondary causes of cognitive impairment; and neuroimaging (brain MRI in all cases apart from two patients who underwent CT because MRI was contraindicated). Seven patients had further CSF analysis and they were all positive for Alzheimer’s biomarkers.

### Perimetry

As required for inclusion in the study, all patients had baseline SHVF. Visual fields were excluded if they exceeded the manufacturer’s cut-off for fixation losses. For the characterisation of the defect, all patients had in addition kinetic Goldmann visual fields (KGVF). SHVF routinely uses a Goldmann size III (4mm^2^) target presented at predetermined locations. Results are based on the threshold for detection of the stimuli, with the output given as mean deviation (MD, that is to say the mean deviation from threshold sensitivity values obtained for each test location in an age-matched control population). In this study, SHVF were performed using Zeiss Humphrey Field Analyzer (*Zeiss Humphrey Field Analyser II I-Series User Manual 2*, n.d.). The protocol used was the 24–2 or, in a few patients, the 30–2, and the test algorithm applied was the Swedish Interactive Threshold Algorithm (SITA).

In the case of KGVF visual targets of a selected size and brightness are moved manually from the periphery toward the centre of the visual field at a constant velocity. The patient’s task is to indicate when they first detect the target moving at, in our protocol, a rate of 10 degrees of visual angle (DVA) per second. A line passing through all visual field locations at which the patient first sees a given target is plotted and referred to as an isopter (a line of equal test luminance and hence equal sensitivity). In this study KGVF were acquired using visual targets moved at a velocity of 10 DVA per second at a luminance of 318 cd/m^2^ and of sizes V4e (64mm^2^), III4e (4mm^2^, which is the same size target as used in the SHVF), and I4e (0.25mm^2^) (30).

In a subgroup of 13/24 patients, additional visual fields were acquired using the Octopus (Haag-Streit 900). Out of these, seven had VF tests more than once and were included in the longitudinal study, although one was not tested with the 10DVA/s target-velocity in the initial assessment.

The Octopus device is automated and permits the use of a range of calibrated target velocities (31). A target size III4e was selected and the velocities employed are given in the relevant test protocol below. At each assessment the vector (stimulus trajectory) was selected randomly by the software.

### Quantification of the visual fields

**Figure 1:**
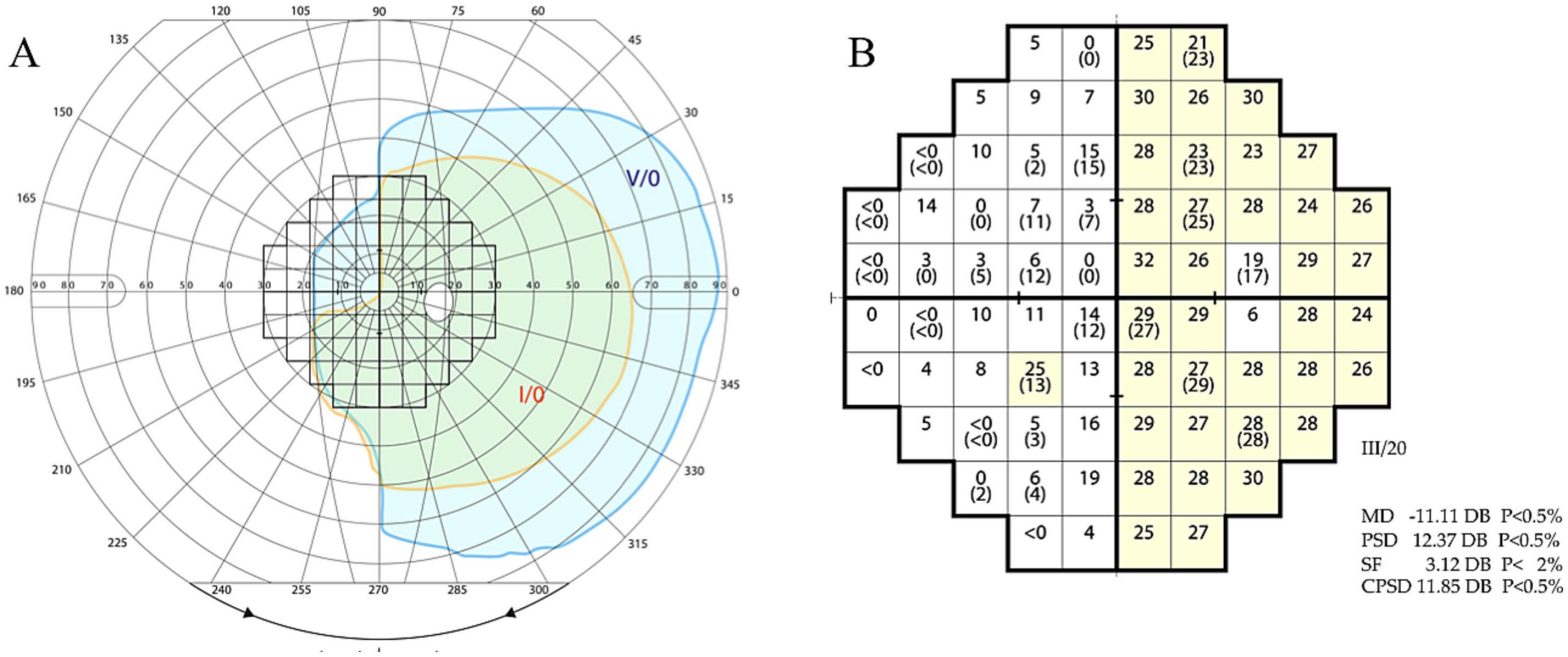
Direct comparison between visual field to kinetic and static stimulation. The grid shown is applied to Goldmann **(A)** where the number of grid squares within the I4e isopter in the affected, left, hemifield are counted (here 11) and Humphrey **(B)** where the number of squares above threshold are counted (here 1). Humphrey output is given as mean deviation (MD), which is the mean deviation from threshold sensitivity values obtained for each test location in an age-matched control population. The calculation is carried out following adjustment for stimulus size and luminance (see Methods). MD, mean deviation. PSD, pattern standard deviation. SF, short-term fluctuation. CPSD, corrected pattern standard deviation. DB, decibel.

One of the aims of the study was to investigate differences in visual performance when either static or kinetic stimuli were employed. Objective comparison between visual fields assessed by KGVF and SHVF is possible after adjustment for size and luminance of the target. Since for human visual perception doubling the size of the stimulus is similar to an increase of 5 decibels (dB) in luminance (31), isopters created with a size I/luminance 0 (0.25mm^2^, maximal luminance) stimulus in KGVF were compared with the points with sensitivity equal or superior to 20 dB to a size III (4 mm^2^) stimulus in SHVF. This comparison was performed using a 30° squared grid for KGVF and for contemporaneous SHVF (maximum three months interval between the two tests). Scores were generated which, in the case of KGVF, corresponded to the number of squares included in the area bounded by the measured isopter (Figure 1A), and, in SHVF, to the number of points with sensitivities above the threshold (Figure 1B). For equalisation of the two tests, when SHVF used the 24–2 setup, which corresponded to a 54-point grid, a 54-point grid was applied to KGVF; if the SHVF setup was the 30–2, the corresponding grid (76-point) was applied to KGVF.

For the purpose of analysing the relationship between the laterality of the visual field loss and the performance in cognitive functions that are known to be lateralised, the ratio between the MD in the right and left hemifields was calculated (Right:left MD ratio - RLMDr), then, in order to reduce skewness of the data, the natural log of this ratio was used (RLMDr log).

Longitudinal data on the initial visual field scores, which represent the measured visual field sizes on KGVF and mean MD per quadrant in the SHVF are available as a Supplementary file.

### Neuropsychological assessment

All patients underwent a comprehensive standardized Neuropsychological battery, encompassing tests for all basic cognitive domains and a more extensive evaluation for posterior cortical functions. Based on the relevant literature, we have considered spelling (32, 33) and calculation (34, 35) as representing left parietal deficits; spatial perception (36–39) and object perception (36, 40–42) as representing right parietal deficits; and shape detection and shape discrimination as reflecting bilateral occipital damage (43). All patients were right-hand dominant. Early cortical visual processing was assessed by tests of shape discrimination [CORVIST (44), Efron squares test (45)] and shape detection (VOSP) (36). For object perception the *Incomplete Letters* and *Object Decision* subtests from the visual object and space perception battery of tests (VOSP) (36) were used, as well as a test of *Unusual Views* (46). For spatial perception, further tests from the VOSP were used (*Dot Counting*, *Position Discrimination, Number Location*, and *Cube Analysis*). Calculation was assessed on the WAIS Arithmetic subtest (47) and Spelling was assessed on the Baxter Spelling Test (32). Memory was assessed using a recognition memory test for words from either the Warrington Recognition Memory Test Battery (48) or the Camden Memory Battery (49). Neglect was assessed by means of a cancellation task (50) and Line Bisection (51). Some patients were also asked to perform the Balloons Test (52).

Missing data occurred due to time constraints or the patient being unable to continue due to fatigue or deterioration in performance.

### Quantification of cognitive deficits

In order to quantify the severity of cognitive deficits a score was developed by an experienced neuropsychologist (MJG) that represented a summary of the available tests for each given domain. Targeted functions were (1) spelling, (2) calculation, (3) object perception, (4) spatial perception, (5) shape discrimination, and (6) shape detection. The total score in the relevant tests for each of domains 1–2 was divided by six, whereas for domains 3–6 total scores were divided by five, thereby creating scores with six (SPEL, CALC,) or five (OP, SP SDISC, SDET) levels of severity, respectively. The aim was to convert results obtained from tests with differing scores (at maximum and at threshold of abnormality) to an equivalent metric. For all these, 0 corresponded to performance within normal limits* and a point was subtracted for each level performed below that expected.

*For spelling and arithmetic, 0 (normal) was given when performance fell within the expected range for the patient’s premorbid IQ. The latter was estimated using the National adult reading test [NART (53); NART-R (54)], which is a standard method for assessing premorbid intelligence (55), and the patient’s educational and occupational achievements For other tests normal limits were as defined in the VOSP (36).

### Statistical analysis

#### Cross-sectional study

To investigate the association between the “cloverleaf-effect” - a measure of reliability of visual fields - and the presence of hemianopia the Spearman’s rank correlation coefficient was used. The Wilcoxon signed rank test was used for the area of the measured visual field comparing static and kinetic stimuli. This test was also used for the comparison of visual fields acquired on the Octopus at two target-velocities (5 and 3 DVA per second). For the correlation of neglect and hemianopia Pearson’s chi-squared test was applied. The latter test was performed in Stata 16. The remaining tests applied in the cross-sectional study were assessed using SPSS 25.

#### Longitudinal study

The aim of this analysis was to model the progression of visual field deficits over time. Individual subject trajectories were plotted for each hemifield (designated as “more” or “less” affected at baseline). A comparison between subjects was also studied. For this purpose, a multilevel mixed-effect model was used (56). In the first level were the hemifields with repeated measures, and in the second level were the hemifields with a single predictor, the hemianopia status (e.g., the more or the less affected hemifield). The same approach was applied to the longitudinal study on cognitive scores, which were set as dependent on time and hemianopia status. These tests were performed in Stata 16.

## Results

### Cross-sectional study

#### Characteristics of the cohort

Table 1 shows demographics, reasons for referral, and cognitive and visual field test findings at presentation. Women were over-represented (16/24) and the mean age at diagnosis was 64 (range: 54–74). All but one patient - who was Turkish - were white British. Five patients had lateralised visual complaints that coincided with the side with greater visual field loss, whereas one (patient 6) complained of right-sided visual loss but was found to have a similar degree of deficit in both hemifields. Four patients (patients 5, 9, 17, 23) were referred after abnormal SHVF were detected at routine examination, even though they had no visual complaint; one out of these had noticed some memory decline over the previous six months.

**Table 1:** Demographics and baseline cognitive and visual fields findings in the 24 patients with posterior cortical atrophy*.

Patient	Sex	Age (y)	Duration (y)**	OP	SP	SPEL	CALC	SDET	SDISC	Neglect***	SHVF		KGVF
											Left	Right	
1	F	61	2–3	-2	-1	N	-2	N	N	L	−2.64	−4.88	Full
2	F	55	1.6	−4	−3	−3	−2	−1	−1	N	−30.51	−17	Inc L> > R HH
3	M	68	2–3	−4	−3	−1	N	N	N	R	−13.09	−3.24	Full
4	M	56	3	−2	−1	-	−3	-	0	N	−19.55	−27.32	Full
55	F	71	0.5	−2	−1	−2	−3	N	N	N	0.39	−16.08	Full
6	F	55	1	−1	−1	−1	−4	N	N	N	−12.4	−12.72	BL inc HH
7	F	67	2	−2	−2	−2	−1	N	N	N	−11.52	−5.05	Full
8	F	73	2–3	−3	−3	−1	−4	N	N	N	−20.19	−2.44	Full
9	M	69		−3	−1	−2	−3	N	N	N	−3.61	−26.46	Full
10	M	61	2–3	−4	−2	N	N	−1	−1	N	−25.23	−13.75	Mild L HH
11	F	66	3	−2	−2	−2	−2	N	N	N	−13.69	−27.83	Inc R HH
12	F	72	2–3	−1	−1	−2	−3	−1	N	L	−29.24	−14.14	Mild L > R HH
13	M	58	1–2	−2	−3	N	N	N	N	N	−13.75	−1.25	Full
													
14	F	66	1–2	−1	−2	N	N	N	N	N	−27.14	−16.31	Full
15	F	54	2–3	−2	−3	−5	−4	−1	−1	N	−6.01	−12.1	Full
16	F	68	1–2	−3	−1	−1	−2	N	N	N	−20.16	−14.67	Full
17	F	74		−1	−1	−3	−4	N	N	N	−15.89	−2.78	Full
18	F	72	2–3	−3	−2	N	N	−1	−1	N	−28.05	−21.99	Mild HH L > R
19	M	57	3	−1	−1	−1	−3	−1	−1	N	1.44	−6.44	Full
20	F	67	1–2	−2	−1	N	−1	−2	0	N	−15.88	−23.95	Full
21	M	65	2–3	−3	−1	−1	−3	−1	N	N	−3.17	−17.16	Full
22	M	57	1	−3	−1	N	N	N	N	N	−13.77	−2.4	Full
23	F	71		N	N	−1	−1	N	N	N	−15.51	−5.16	Full
24	F	55	1–2	−1	−1	N	N	N	N	N	−17.19	−3.19	Full

For cognitive deficits, numbers correspond to a score created to represent a summary of the tests for each given domain. The total score in the relevant tests for SPEL and CALC was divided by six, whereas for OP, SD, SDISC and SDET total scores were divided by five, thereby creating scores with six (SPEL, CALC) or five (OP, SP, SDISC, SDET) levels of severity, respectively. For all these, 0 corresponded to performance within normal limits, and a point was subtracted for each level performed below that expected. For SPEL and CALC, 0 (normal) was given when performance fell within the expected range for the patient’s premorbid IQ. *The three patients with neglect had line bisection deviation toward the opposite side of the neglect. SHVF, static humphrey visual field; KGVF, kinetic goldmann visual field; OP, object perception; SP, spatial perception; SPEL, spelling; CALC, calculation; SDISC, shape discrimination; SDET, shape detection; Y, years; HH, homonymous hemianopia; N, normal/not present; L, left; R, right; BL, bilateral; Inc, incongruous.

*A more extensive version of this table is available in the supplementary material. **Duration of symptoms prior to presentation.

#### Characteristics of the visual fields at baseline

**Figure 2:**
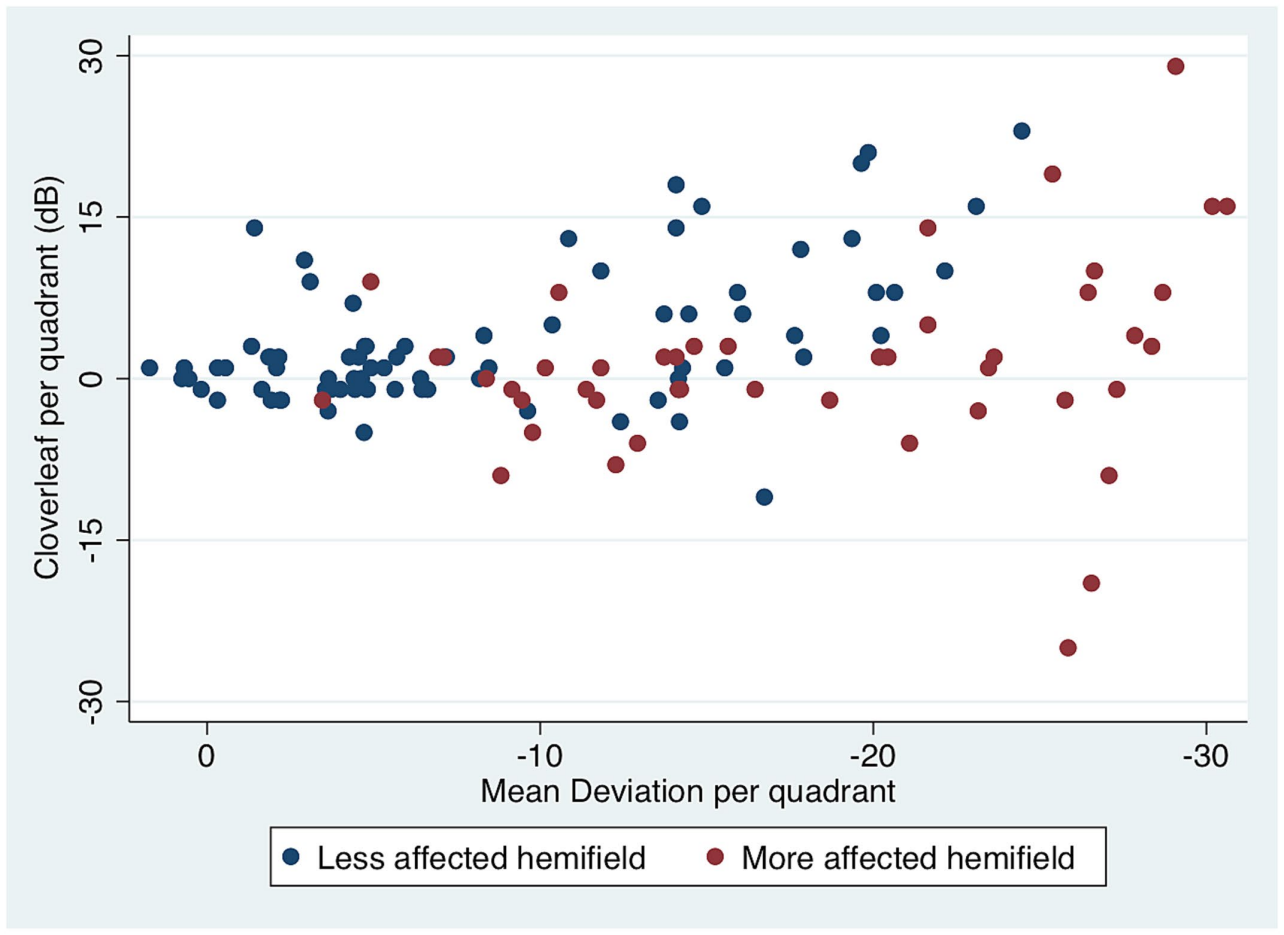
Test reliability according to visual field loss (mean deviation) in 24 patients with PCA. An increase in variability in response is shown in relation to the overall deficit. The detection threshold for a point tested at the start of the test is compared with an immediately neighbouring point tested subsequently. Each data point represents the differences (“cloverleaf”) between these two visual field points, in decibels (dB). The responses predominantly show a false negative effect (an increase in threshold on subsequent test indicating a deterioration in performance: the “cloverleaf” effect) with some evidence of false positive (a reduction in threshold on subsequent test). The measure of cloverleaf per quadrant increases with a lower overall visual sensitivity in that quadrant (Spearman’s rank correlation coefficient, rho = −0.22, *p* = 0.008). Results are shown for the less (blue) or more (red) affected hemifields, respectively.

To check the reliability of the visual field test results a localised measure was employed based on the “false negative index” of the SHVF protocol (the “cloverleaf” effect (57)). The measurement was carried out independently in each quadrant of the VF at the initial testing session only. Threshold detection of the first point tested in each quadrant was compared with the threshold for an adjacent point tested subsequently in the same test session. This quantifies a positive or negative deviation in the detection threshold at the tested locations. Using Spearman’s rank correlation coefficient, there was an inverse correlation between this measure and overall MD for each quadrant (Figure 2). Thus a lower sensitivity was accompanied by a reduction in overall reliability as evidenced by a greater occurrence of false negative and false positive responses (predominantly false negative, i.e., performance deteriorated as the test progressed). In addition, a greater degree of incongruity between homonymous VF (judged by visual inspection) was seen in cases having an overall greater deficit.

**Figure 3:**
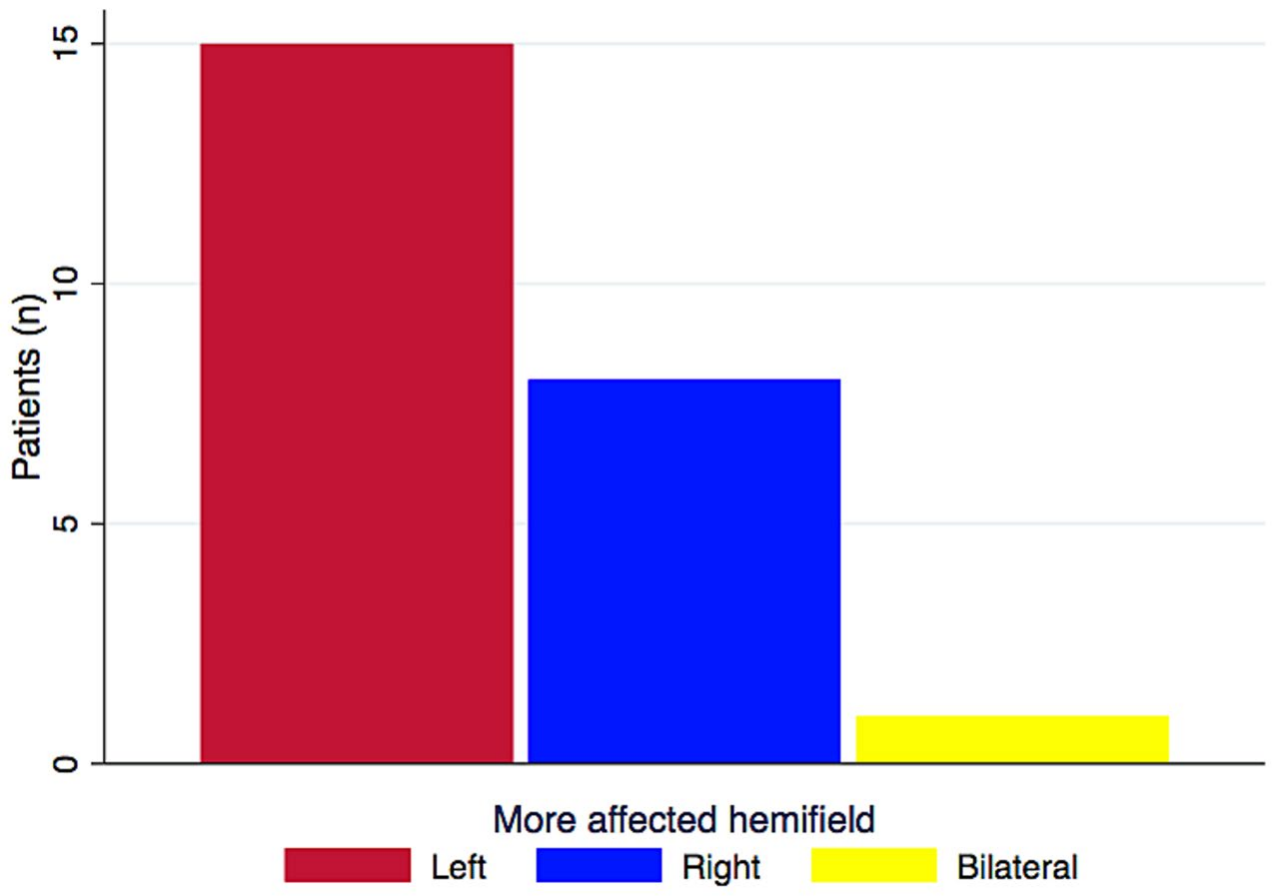
More affected hemifield in 24 PCA patients with HH. HH was predominant on the left in 62.56% (*n* = 15) of the patients. One patient had a bilateral symmetrical HH. (PCA, posterior cortical atrophy; HH, homonymous hemianopia). In a paired *t*-test, the difference between left and right showed a trend (*t* = 1.4968, df = 23, *p* = 0.074).

At initial assessment the left was the greater affected or the only affected hemifield in two-thirds of the patients. Differences between left and right-predominant HH confirms a trend (*t* = 1.4968, *p* = 0.074) (Figure 3).

Visual hemi-neglect was observed in three patients (2 left, 1 right; Table 1). Pearson’s chi-squared test was applied to compare the laterality of HH and neglect. There was no evidence of dependence [X(1) = 1.17, *p* = 0.28 on the left, and X(1) = 0.58, *p* = 0.46 on the right].

**Figure 4:**
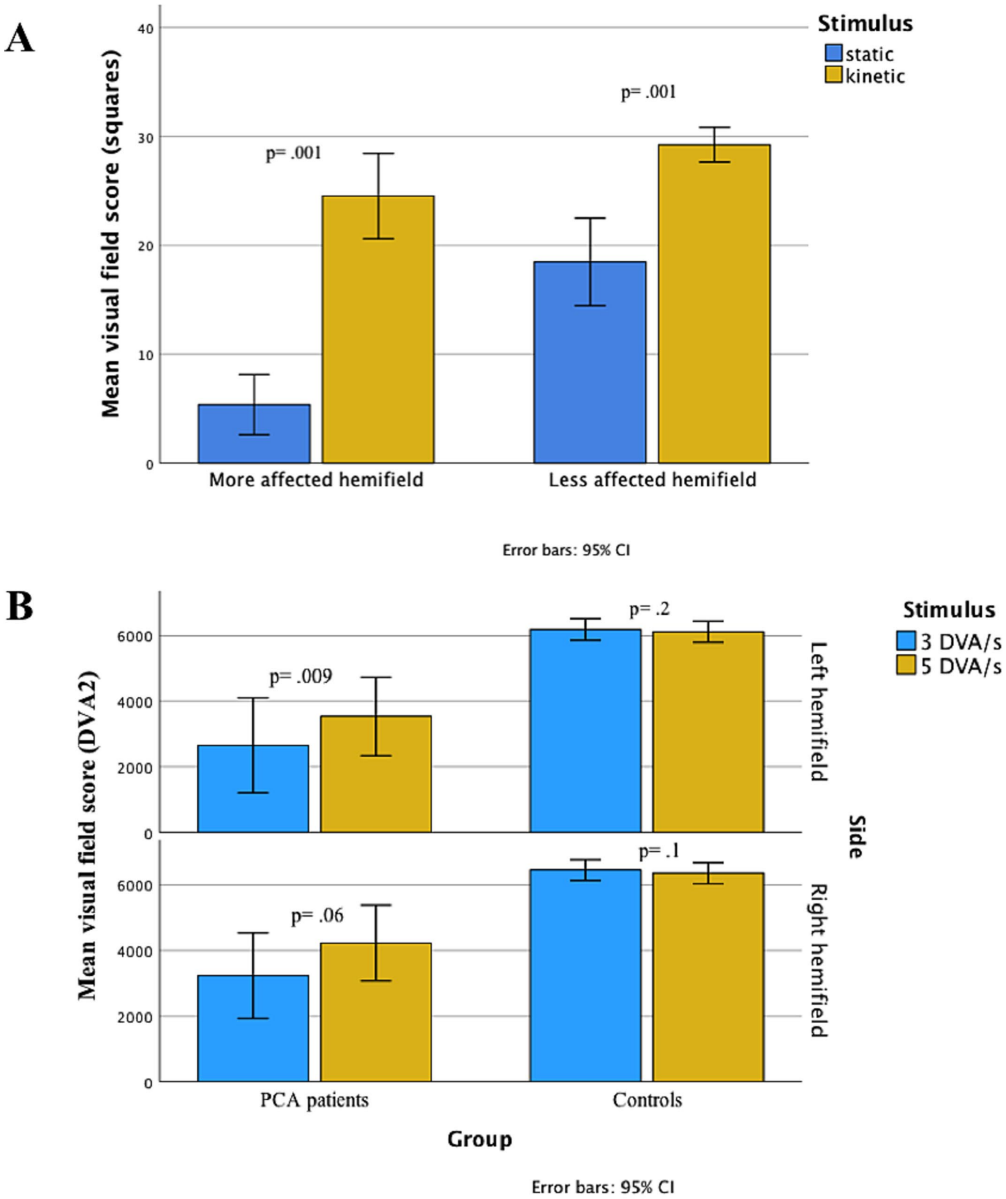
Differences in means of visual field size to static and kinetic testing at baseline. **(A)** Visual fields to kinetic (KGVF) and static (SHVF), measured with equivalent targets in 24 patients with PCA. Velocity of stimulation in KGVF testing was 10 deg./s. When visual field sizes were compared using Wilcoxon signed ranks test, statokinetic dissociation was significant in both less and more affected hemifields (*Z* = −4.289, *p* = 0.001; and *Z* = −4.288, *p* = 0.001, respectively), but greater magnitude in the more affected hemifield (*Z* = −3.287, *p* = 0.001). The number of squares in the vertical axis corresponds to the size of the visual fields measured with a squared grid applied to both KGVF and SHVF, as detailed in the text. **(B)** Visual field dimensions as acquired with 5 DVA/s (blue) or 3 DVA/s (red) target-velocity, in the left (upper panel) and right (lower panel) hemifields in 13 PCA patients and 30 healthy controls. Wilcoxon signed ranks test was used to compare means. Whereas in PCA patients, visual fields obtained with 3 DVA/s target-velocity were smaller than visual fields to the 5 DVA/s target-velocity in both left and right hemifields (the difference was significant in the left (*p* = 0.009) and a trend was observed in the right hemifield (*p* = 0.06)), no difference between visual fields acquired with the these two target-velocities was observed in controls. PCA, posterior cortical atrophy. KGVF, kinetic Goldmann visual field. SHVF, static Humphrey visual field. DVA, degrees of visual angle.

Stato-kinetic dissociation (the Riddoch effect; SKD) was observed in all affected visual fields, with the defect measured by KGVF being less than that observed with SHVF in all cases (Table 1). Mean differences between SHVF and KGVF were larger in the more affected hemifield (Figure 4A). In thirteen patients who had visual fields acquired on the Octopus using three target velocities, dissociation was observed in favour of a greater defect when the slow target velocity was applied. Such dissociation was not observed in healthy controls (Figure 4B).

### Longitudinal study

#### Change in SHVF with disease progression over time

**Figure 5:**
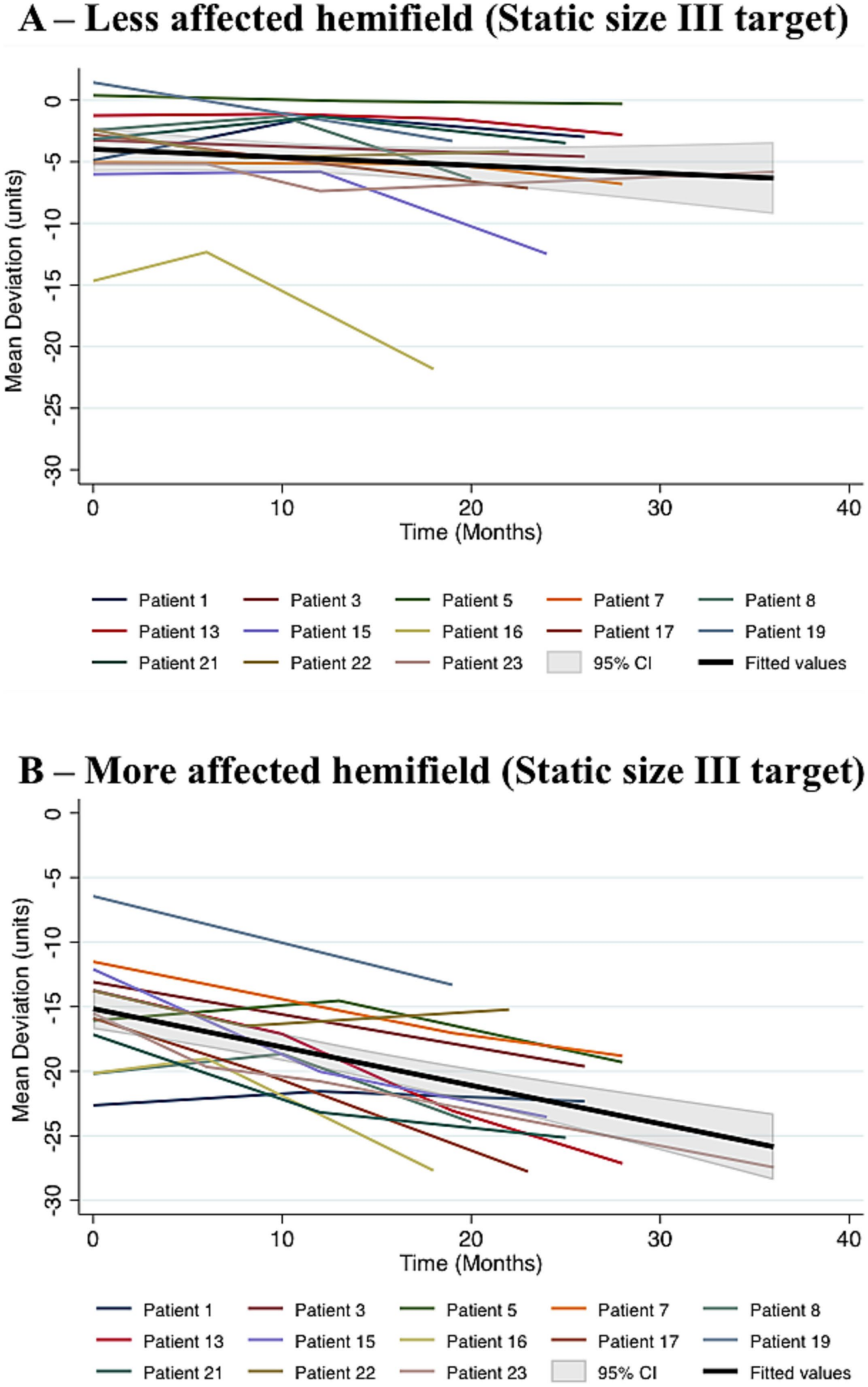
Visual fields to static testing over time in 13 patients with PCA. Patients included in this analysis (13/24 PCA patients) had relatively mild hemianopia to SHVF at baseline. Progression in the less **(A)** and more affected **(B)** hemifields was analysed using mixed-effects multilevel regression. MD declined at a rate of −0.09 units/month (*p* = 0.007) in the less affected hemifield, which was significantly lower than the rate of decline in the more affected hemifield (−0.29 units/month, *p* = 0.001). SHVF, static humphrey visual field test. PCA, posterior cortical atrophy.

Twenty patients had SHVF acquired at least twice and nineteen were initially included in this study – one having a symmetrical HH was excluded. Duration of follow-up ranged from six to 42 months. Because of a potential floor effect, six patients who entered the study with very low MD in at least one hemifield - which prevented detection of any further change - were excluded from the present analysis. A cut-off for inclusion was established at MD > −23 units in the two hemifields. For the purpose of this analysis, the maximal follow-up duration was set to 36 months, in order to select the epoch in which change was still occurring. Only five patients had time-points after this and, by then, visual loss had reached the floor in at least one hemifield in all of them. In the patients retained for this analysis (*n* = 13), during this interval, the rate of progression of MD was −0.09 units/month (*p* = 0.007) in the less affected hemifield and −0.29 units/month (*p* = 0.001) in the more affected hemifield. The difference in rate of progression between hemifields was statistically significant (*p* = 0.001) (Figure 5).

#### Change in KGVF over time

**Figure 6:**
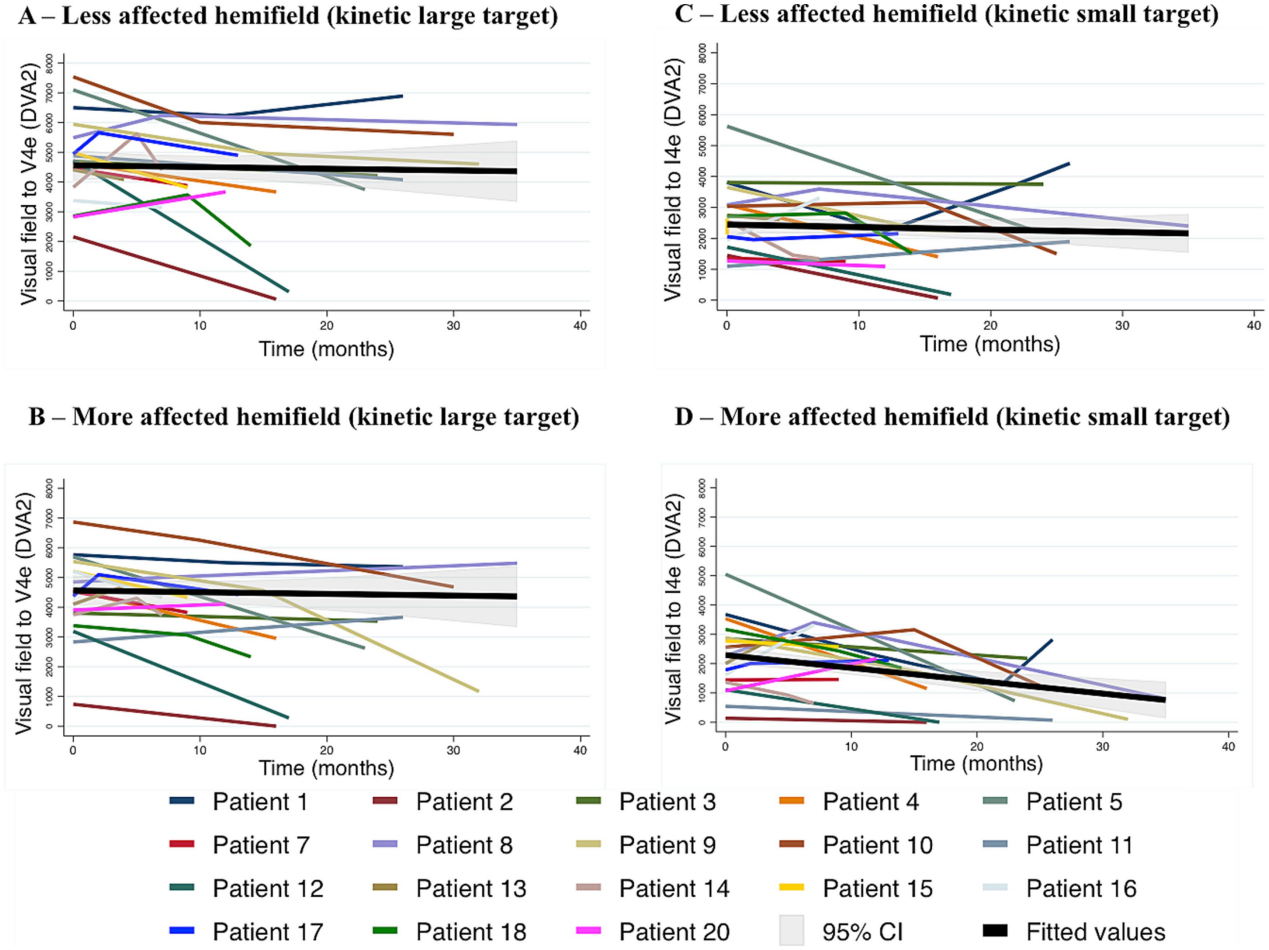
Visual fields to kinetic testing over time in 18 patients with PCA, using two target-sizes. **(A,B)** Loss to kinetic testing with a V4e target in the less **(A)** and in the more **(B)** affected hemifield, as defined by static loss at baseline. In a mixed-effects multilevel regression analysis visual field size declined 40.19 DVA^2^ per month (*p* = 0.008) in the former, whereas in the latter the rate of progression was −50.64 DVA^2^/month (*p* = 0.001). The difference in rates of decline between the two hemifields group did not reach statistical significance (*p* = 0.15). **(C,D)** Visual fields as acquired with the I4e target in the less affected **(C)** and in the more affected hemifield **(D)**. In the mixed model analysis, rates of progression were −26 DVA^2^/month (*p* = 0.03) and −52 DVA^2^/month (*p* = 0.001), respectively. When these rates were compared by the addition of an interaction term between time and the relative degree of hemianopia, decline was significantly faster in the more affected hemifield (*p* = 0.02). PCA, posterior cortical atrophy. DVA, degree of visual angle. DVA^2^, squared degree of visual angle.

Eighteen patients had KGVF acquired on two or more occasions, using the V4e and I4e targets at 10 DVA/s speed of stimulation, and were included in this analysis (Figures 6A,B). Visual fields to V4e target declined in both the less and more affected hemifields, in the former at −40.19 DVA^2^/month (*p* = 0.008) and in the latter at −50.64 DVA^2^/month (*p* = 0.001). When these rates were directly compared by means of an interaction term between time and hemifield added to the mixed effect ML regression, no statistically significant difference was observed between them (*p* = 0.15), whereas every month elapsed accounted for a decline of 34.49 DVA^2^ (*p* = 0.01) (Figure 6A). Figure 6B illustrates the variation of the corresponding visual field size over time in the less and in the more affected hemifields to the I4e target. In the former, every month passed meant a decrement of 26 DVA^2^ (*p* = 0.03) in the visual field, whereas the rate of progression in the latter was −52 DVA^2^ /month (*p* = 0.001). In the analysis of the interaction between time elapsed and hemifield, the difference between the two rates reached statistical significance (*p* = 0.02).

#### Change in visual fields to kinetic testing at three different velocities of stimulation over time

##### 10 DVA/s

**Figure 7:**
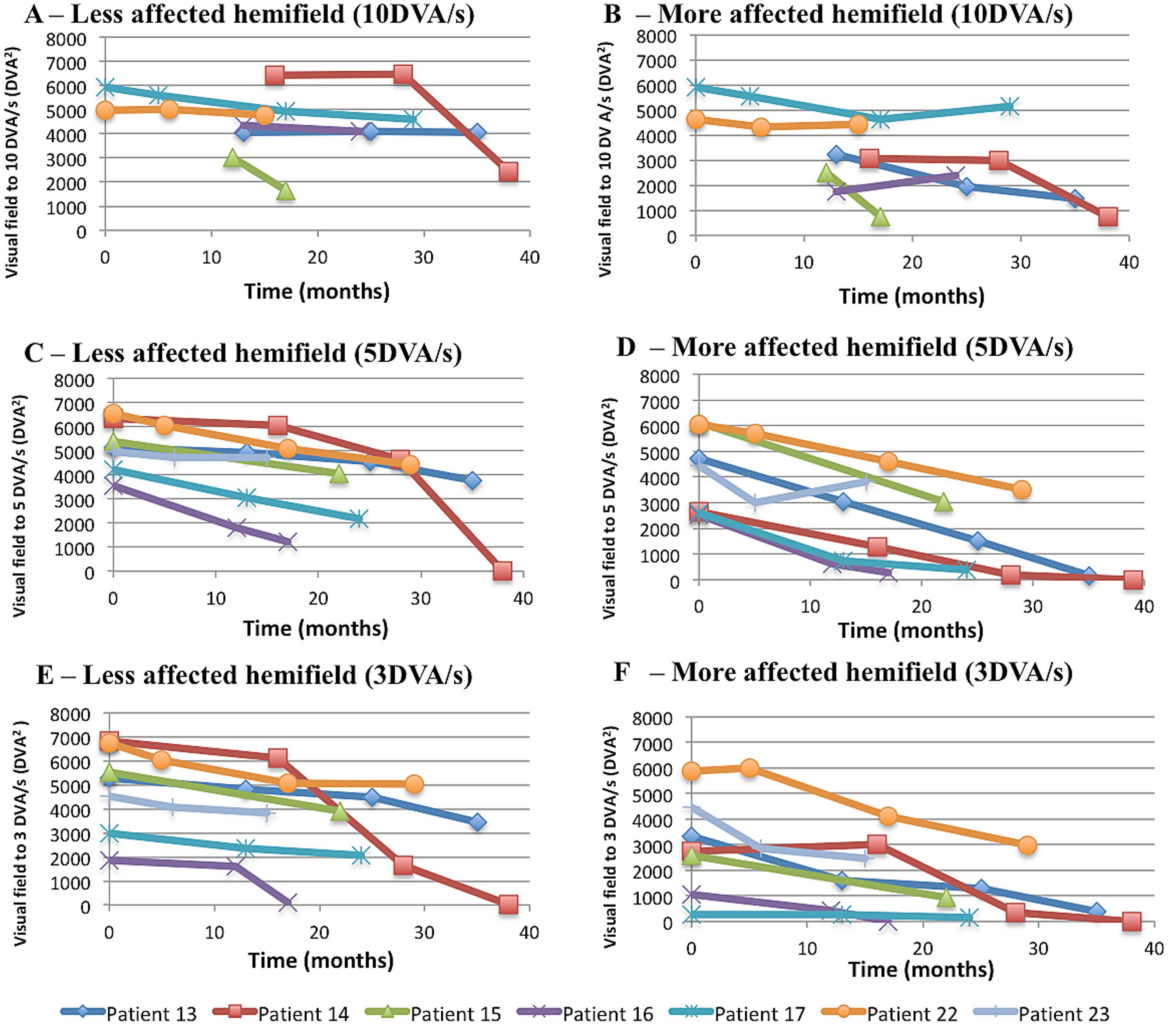
Visual fields to kinetic testing at three different target-velocities. **(A,B)** Visual fields acquired with 10 DVA/s target velocity in the less **(A)** and more affected hemifield **(B)**. The rate of progression was −54 DVA^2^/month (*p* = 0.02) in the latter and −81 DVA^2^/month (*p* = 0.001) in the former. A mixed effects regression analysis with interaction between time and relative degree of hemianopia showed that the difference in rates of progression between the less and the more affected hemifield was not statistically significant (*p* = 0.411). **(C,D)** Visual fields acquired with 5 DVA/s target velocity in the less **(C)** and more affected hemifield **(D)**. When analysed with mixed-effect model regression, rates of progression were −85 DVA^2^/month (*p* = 0.001) in the former and −111 DVA^2^/month (*p* = 0.001) in the latter. The difference in rate of progression was not statistically significant (*p* = 0.278). **(E,F)** Visual fields acquired with 3 DVA/s target velocity in the less **(E)** and more affected hemifield **(F)**. In a mixed-effect model regression analysis, visual fields deteriorated at the rate of −92 DVA^2^/month (*p* = 0.001) in the former and −87 DVA^2^/month (*p* = 0.001) in the latter. In a mixed effects regression analysis with interaction between time and relative degree of hemianopia, rates of progression in the less and more affected hemifield were not statistically different (*p* = 0.756). In all graphs, each time point represents one assessment. DVA, degree of visual angle. DVA^2^, squared degree of visual angle.

When acquired with a 10 DVA/s target-velocity, visual field sizes did not statistically differ between less and more affected hemifields, although numerically the former exceeded the latter (difference 763 DVA^2^ in favour of the less affected hemifields, *p* = 0.216). Deterioration occurred in both the less and more affected hemifields, at the rates of −54 DVA^2^/month (*p* = 0.02) and 81 DVA^2^/month (*p* = 0.001), respectively. These rates of decline were not statistically different when directly compared (difference of 22 DVA^2^ in favour of the more affected hemifield, *p* = 0.411) (Figures 7A,B).

##### 5 DVA/s

When visual fields were acquired with a 5 DVA/s target-velocity, the less affected hemifield exceeded the more affected hemifield in size (difference = 1,229 DVA^2^, *p* = 0.005). The rate of deterioration was numerically higher in the more affected hemifield (111 DVA^2^/month versus 85 DVA^2^/month, *p* = 0.001 for both), however the difference between the rates was not statistically significant (*p* = 0.278) (Figures 7C,D).

##### 3 DVA/s

Assessed with a 3 DVA/s target-velocity, visual fields were smaller in the more affected hemifield (difference = 1881 DVA^2^, *p* = 0.001). The rates of progression were −92 DVA^2^/month (*p* = 0.001) and −87 DVA^2^/month (*p* = 0.001) in the less and more affected hemifields, respectively, which were not statistically different when compared by means of an interaction term between time and hemifield (rate of decline in the less affected hemifield exceeded numerically the more affected hemifield by 6.4 DVA^2^/month, *p* = 0.756) (Figures 7E,F).

#### Relationship between HH and cognitive deficits

**Figure 8:**
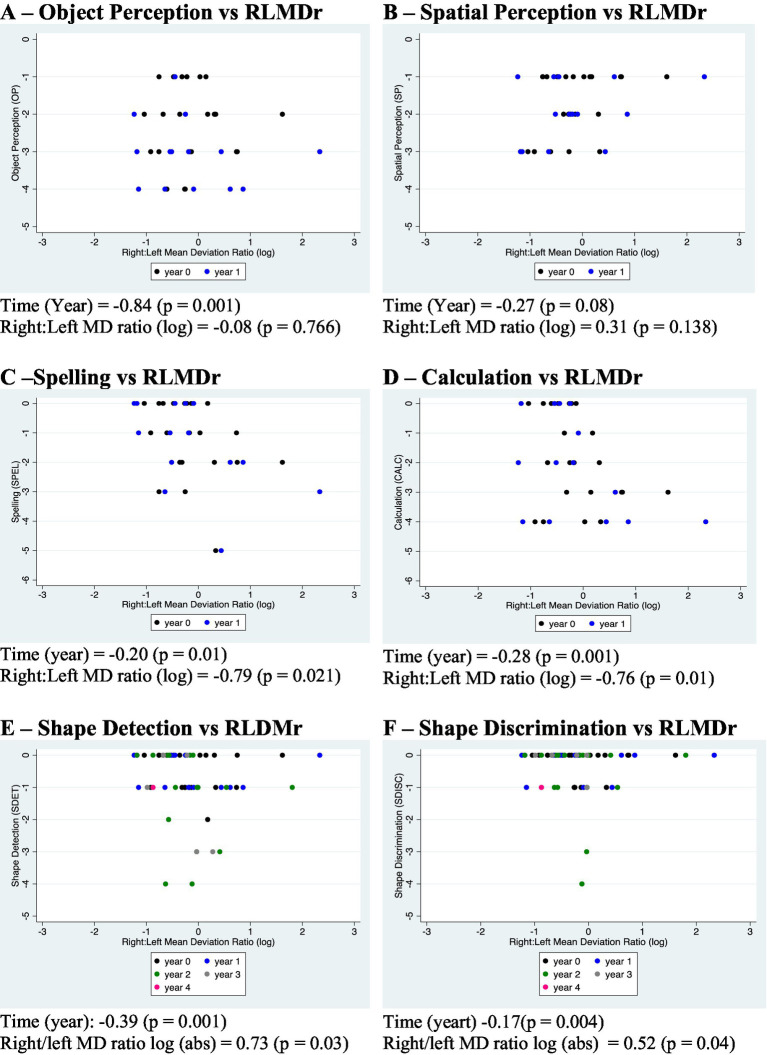
Longitudinal associations between the laterality of the hemianopia and cognitive deficits. **(A–D)** A comparison is shown between the degree of lateralisation of the visual field deficit (log Right:Left MD ratio, horizontal axes) and four potentially lateralised cognitive deficits [object perception **(A)**, spatial perception **(B)**, spelling **(C)**, and calculation **(D)**, vertical axes] in 21 PCA patients through one year of follow-up. Mixed model linear regression was used to analyze cognitive scores according to time and the Right:Left MD ratio log. There is a statistically significant correlation between cognitive scores and time elapsed in three measurements but not spatial perception. There is an association between cognitive deficits over time and the degree of asymmetry in the visual field deficit in spelling and calculation – i.e., deficits related to the left hemisphere -, but not in object perception and spatial perception, functions that disparately rely on the right hemisphere in comparison to left. **(E,F)** Comparison between the log Right:Left MD ratio and two deficits related to bilateral occipital damage [shape detection **(E)** and shape discrimination **(F)**] through up to five years of follow-up. There is an association between longitudinal scores for both functions and time elapsed, as well as between these scores and the absolute value of the RLMDr log. Shape detection and shape discrimination scores decrease as the absolute value of log of RLMDr approaches zero, i.e., the visual field deficit is more symmetric. Data points represent scores of individual patients at different years, which are designated by different colours. OP, SP, SPEL, and CALC are scores derived from the summary of relevant tests for object perception, spatial perception, spelling, and calculation, respectively. In each of them, a score of zero means performance within normal. Performance at the bottom is −5 for OP and SP and −6 for SPEL and CALC.

For the longitudinal analysis of lateralised functions (Figures 8A–D), only results up to one year of follow-up were included, given the increasing occurrence of a floor effect in cognitive scores (especially in spelling and calculation) and the complex dynamic of the HH ratio after this period - with the development or worsening of HH in the second hemifield, more so if a floor effect appeared in the first affected hemifield, the direction of change of the Right:Left MD ratio (RLMDr) inverted; after this point, the laterality of the neurodegenerative process could still be reflected on the RLMDr but not on its change. In contrast, in the longitudinal analysis of shape discrimination and shape detection – deficits reflecting bilateral occipital damage (Figures 8E,F), an initial ceiling effect was observed. All but one patient (patient 7, whose initial RLMDr was 1 and remained so all along follow-up) had disproportionate HH, which tended to become bilateral with time. Given this, all time points available were included in this analysis.

Twenty-one out of 24 PCA patients had full neuropsychological assessment and contemporary (within three months) visual field test and could be included in this investigation.

There was no significant longitudinal relationship between scores in object perception and the RLMDr log (coefficient −0.08, *p* = 0.766) and between spatial perception and the RLMDr log, although a trend could be observed toward a positive association between the RLMDr log and spatial perception (coefficient 0.31, *p* = 0.138). The RLMDr log had a negative association with scores in Spelling (coefficient −0.79, *p* = 0.021) and Calculation (coefficient −0.76, *p* = 0.01) (Figures 8A–D).

A positive longitudinal association was observed between the absolute value of the Right:Left MD ratio log and scores in shape detection (coefficient = 0.73, *p* = 0.03) and shape discrimination (coefficient = 0.52, *p* = 0.04). As the absolute value of the Right:Left MD ratio log decreased to 0 (i.e., the ratio approached 1), scores in these functions declined (Figures 8E,F).

## Discussion

### HH in PCA patients is a primary deficit

The study reported here represents the largest cohort of patients with PCA presenting with HH thus far. PCA patients are likely to produce unreliable visual field tests, since coexisting visuo-spatial deficits may lead to fixation losses and false negative or false positive responses. Visual field tests with excess fixation losses were excluded by manufacturer standards. With respect to false negative responses, we have considered whether this finding in the present cohort reflects visual fatigue, as is usually the case (57), or if their occurrence could be related to the HH. Indeed, when a measure of false negatives (the cloverleaf effect) was studied across regions of the visual field (Figure 2), an inverse correlation between this index and MD in that region (quadrant) emerged, indicating that this type of error was related to the severity of the regional visual field loss, not a global effect of performance across the cohort. Such a relationship has been observed with visual field loss from other causes, e.g., glaucoma (58).

Having demonstrated a sufficient degree of reliability in the visual field performance for serial comparison, an important question is whether the measured defect could reflect a higher-level attention disorder rather than loss of threshold detection *per se* (12). Indeed, in primary position of gaze, where hemiretinal and hemispatial planes coincide, a hemifield loss may result from neglect (59). However, only three patients (12%) had evidence of neglect on standard testing and the lateralization of neglect and of HH were not dependent. It is unlikely that the measured field defect was a consequence of neglect. Other characteristics of the visual field deficits are difficult to account for on the basis of neglect, such as non-congruity and regional variation in the deficit within a hemifield (e.g., inferior quadrantanopia). We are aware that vertical neglect has rarely been observed in PCA (60), however, in this study, no patient displayed vertical neglect in the cancellation task.

It is also worth noting that in the patients with both neglect and HH no interaction was observed. It is known that, in line bisection tasks, patients with hemi-neglect tend to displace the midline to the hemifield away from the affected side, whereas patients with HH show midline deviation toward the affected hemifield (61); in patients with hemi-neglect and HH arising from the same hemisphere – a rather common occurrence in the stroke setting – the opposing line deviations may cancel-out, however the interaction works in favour of worse performance in the affected hemifield (62). In the present study, the three patients with neglect, including the two in whom the two deficits arose from the same hemisphere, showed midline deviations expected on the basis of the neglect only. It should also be noted that the cited studies were carried out on patients with dense hemianopia, not *relative* loss as in the PCA cases and the lack of a lateralized abnormality in performance on line bisection is compatible with the hypothesis that neglect does not play a part in the pathogenesis of HH.

### Left-sided HH predominated in PCA patients

In this cohort, left-sided HH was found more frequently than right-sided HH (16/24) (Figure 3). Indeed, the majority of the cases published reporting HH in PCA patients illustrate left HH (20–23, 28, 63). It seems likely that the clinical criteria for diagnosis of PCA give rise to biases toward identifying patients with left HH. Greater involvement of the right hemisphere has consistently been reported in imaging studies of PCA patients (8, 25, 64, 65) The favoured explanation for this is that the cardinal features of PCA - i.e. visuospatial deficits - are right-hemisphere deficits (42, 66). The predominance of left HH therefore suggests that visual field deficits develop in a continuum with cognitive deficits. This hypothesis may be tested by comparing the laterality of visual field deficits with lateralised cognitive deficits. An interesting comparison would be with logopenic progressive aphasia. However visual field defects are uncommon in that syndrome although an exhaustive study has not been carried out to our knowledge (67).

### HH in PCA is characterised by statokinetic dissociation

At baseline, the perimetric deficits to kinetic stimuli strikingly differ from those acquired with a static stimulus (Table 1). Although (according to inclusion criteria) all patients had homonymous visual field defects to SHVF, two thirds had full KGVF. Statokinetic dissociation (SKD) was greater in the more affected hemifield (as defined by SHVF) (Figure 4A), suggesting that SKD increases in magnitude with an increase in the overall deficit, due to a more rapid deterioration in performance to static than kinetic stimuli.

SKD can occur in visual field loss from a variety of causes (68), as well as in healthy subjects (69), however in these contexts, as in most cases seen in clinic, it is typically small, often measuring only a few degrees of visual angle (70). The substantial SKD observed in PCA resembles the original description of the Riddoch phenomenon (26) and, we suggest, might reflect a characteristic of the underlying pathology. Riddoch first drew attention to the sparing of kinetic detection within homonymous visual field loss due to traumatic occipital lobe lesions. Subsequently, SKD was considered to be pathognomonic of visual cortical lesions, however this was dismissed after the phenomenon was also observed in lesions of the anterior visual pathways (71). An intrinsic greater resistance of kinetic detection to diminished visual input was then postulated as the most likely explanation, although a dissociation at the level of the magnocellular and parvocellular input to the lateral geniculate nucleus was postulated in cases of chiasmal compression (72). Further studies are required to elucidate other aspects of the residual vision other than simple detection of a moving target – for example velocity discrimination and motion coherence studies – so that comparison can be made with other descriptions which have been likened to the Riddoch phenomenon in cases of damage to visual cortex (73–75). In such cases it has become clear that the likely origin of the Riddoch phenomenon is the preservation of direct projections from the LGN to cortical area V5. This may involve both ipsilateral and contralateral projections (75). Whether this is the case with PCA is unknown.

Remarkably, when VFs were acquired using two different target-velocities (3 DVA/s vs. 5 DVA/s), patients with PCA exhibited low-high-velocity dissociation, with smaller VFs to 3 DVA/s compared to 5 DVA/s target-velocity, while no such dissociation was found in healthy controls (Figure 4B).

### Progression of visual fields

In the longitudinal study SHVF deteriorated in all patients, more rapidly in the more affected hemifield. In some cases only after the HH had reached the “floor” would the other hemifield start deteriorating. An interhemisphere comparison was problematic due to this phenomenon. However, when patients who had reached the “floor” in at least one hemifield were excluded, a consistent pattern of deterioration was seen, namely a greater rate of deterioration in the more affected hemifield (Figure 5).

By contrast, while SHVF progressed steadily, KGVF (manual stimulation at 10 DVA/s) visual fields showed a highly variable course, with some undergoing little or no deterioration, while others had marked decline (Figure 6A). This behaviour was not common in the case of SHVF (Figure 5). In addition, in comparison with SHVF, deterioration in KGVF was not as well lateralised. As a rule, when one hemifield deteriorated, so did the other. Accordingly, rates of progression to the V4e target-KGVF did not significantly differ between the less and more affected hemifields. This could reflect the deficit appearing at a more advanced stage, when disease is known to become more symmetrical (29). When the smaller target I4e was used instead, a greater number of patients and hemifields deteriorated and the rate of decline was significantly greater in the initially more affected hemifield (Figure 6B). This suggests that deterioration in kinetic testing is lateralised and progresses in a stereotyped sequence following deterioration to static. Threshold detection of the V4e-target is neither sensitive enough to detect an early deterioration over time, nor to show differences between hemifields.

Since a smaller target is more sensitive to demonstrating deficit laterality, we studied whether the velocity of stimulation may behave similarly. A subgroup of 13 patients had KGVF acquired on the Octopus, using different target-velocities (and stable target-size). In contrast to KGVF at 10 DVA/s (Figures 7A,B), decrement occurred in all patients and hemifields when the velocity of stimulation was 5 DVA/s (Figures 7C,D) or 3 DVA/s (Figures 7E,F), i.e., more similar to SHVF. Therefore, the initial relative preservation of visual fields to kinetic testing is related to target velocity. In regard to laterality, no statistically significant difference was observed between the rates of progression in the less and more affected hemifields with either 3 DVA/s or 5 DVA/s target-velocities; nonetheless, visual field area to both target-velocities was reduced from baseline in the more affected hemifield confirming that deterioration to kinetic begins in the hemifield with the greater static deficit.

Altogether, these observations suggest that, in PCA patients, visual field loss to static and kinetic stimuli represent two stages in the progression of the same, lateralized, pathology. We propose two mechanisms whereby the disease stage would determine SKD: (1) a quantitative hypothesis: given the intrinsic greater resistance of motion detection to diminished visual input (71, 72), at a given stage of disease, the number of cells remaining in the relevant visual pathway is sufficient to allow kinetic stimulus to be detected but not static; and (2) a qualitative hypothesis: at a given stage of disease, AD pathology disproportionately affects superficial layers of the striate cortex, leaving relatively spared the deeper cortical layers, which harbor the cells involved in motion detection (76). The latter hypothesis is supported by the known hierarchical distribution of AD pathology through the visual cortex demonstrating preferential involvement of superficial layers (77, 78). The finding that deterioration of kinetic detection follows a gradient of velocities further suggests that this deficit reflects the severity of the neurodegenerative process in a continuous fashion and could favour the quantitative hypothesis. In hemianopic PCA patients, visual cortical input is diminished, as evidenced by the lateralised degeneration of the optic radiations (25); furthermore, motion sensitive neurons in the peripheral visual field representation in V1 generally prefer moving stimuli in the higher velocity range (79). One possibility is that there is a stage in the process of degeneration where input is sufficient for detection of a higher but not a lower-velocity target.

### The relationship between HH and cognitive deficits

Previous attempts to classify PCA phenotype have proposed up to four variants – a dorsal stream, a ventral stream, a dominant-parietal, and a primary visual (bi-occipital) variant (7, 15, 80, 81). Under this approach, HH is expected to occur, along with basic visual deficits, in the primary visual variant, which is considered rare (15, 81). However, a study that defined the PCA primary occipital variant on the basis of disproportionate occipital hypometabolism (in relation to occipitotemporal or occipitoparietal) observed no difference in the prevalence of HH (about 50%) between this and the other variants, although the method of visual field testing was not given (82). This illustrates the difficulty situating HH within this classification framework. In the cohort presented herein HH occurred at stages when visual cortical deficits reflecting bi-occipital damage (shape detection, shape discrimination) were not detected, whereas higher order deficits, such as (spatial perception and visual object perception deficits) were present. Furthermore, in all except one patient HH showed asymmetry. We therefore tested the hypothesis that HH lateralization would show a correlation with the predominant lateralization of the cognitive functions tested.

At baseline, comparison of the RLMDr and cognitive function scores suggested an association of right HH with impaired spelling and calculation (i.e., left parietal functions), and between left HH and deficits in spatial perception, although correlations did not reach significance at baseline (Not shown). Progression of deficits over the following year confirmed the trend in regard to left parietal functions only: the greater the right hemifield deficit, the greater was the deterioration in spelling (coefficient −0.79, *p* = 0.021) and calculation (coefficient −0.76, *p* = 0.01). By contrast, performance in object perception and spatial perception did not show a correlation with HH lateralisation (Figures 8A–D).

These results suggest that the laterality of the HH does relate to the cognitive profile. Most significantly, patients with right-sided HH are more likely to be impaired in Spelling and Calculation, i.e., reflecting a greater dysfunction of the left hemisphere, thereby suggesting the primacy of the hemisphere in disease progression. A trend appeared in baseline for a correlation between left-sided HH and greater impairment in spatial perception (coefficient 0.31; *p* = 0.1), but progression in spatial and object perception were not affected by the laterality of the HH in the longitudinal study.

The lack of correlation between right parietal functions and the pattern of HH deserves some consideration. Firstly, OP and SP were impaired in all patients from baseline, therefore it is no surprise that these measures do not reveal a subgroup of PCA patients. The clinical definition of PCA requires a cortical visual deficit (15, 19), which is the preferred explanation for the demonstration on imaging studies of PCA as a posterior cortical degeneration biased toward the right-hemisphere (10, 12, 64, 65, 82–84). An alternative explanation may lie in the fundamental correlates of OP, which may not be as well lateralized as those of Spelling and Calculation, so that the effect may not be detected in a small sample. Indeed, while several studies support a predominant role of the right hemisphere in the perceptual stage of OP (36, 40–42, 46, 66), others have suggested that at least some aspects of OP depend on integrative function between right and left hemispheres (38, 39). However, since the controversy in regard to laterality generally does not apply to Spatial Perception, the most likely explanation for the different relationship between HH and right and left hemisphere functions is the bias of the PCA definition toward right hemisphere dysfunction. Notwithstanding this bias, the findings support HH as progressing in parallel with cognitive deficits following a primacy of the hemisphere, which was already anticipated by the predominance of left HH in this cohort.

The association between visual field findings and cognitive deficits was further tested in relation to deficits known to be associated with bilateral occipital damage. Remarkably, basic visual functions (shape discrimination and shape detection) were initially preserved in 58% (14) of the patients, but deteriorated in the majority, with greater impairment correlating with the development of bilateral hemianopia (Figures 8E,F).

In summary, our findings indicate that the pattern of HH is related to the cognitive profile in PCA patients. Any attempt to comment on differences in the cognitive profile between PCA patients with and without HH is bedevilled by the fact that in our series all patients have presented with a visual field deficit, and in the majority of cohorts there have been no perimetric studies. HH may be missed in cognitive clinics where VFTs are not routinely performed, more so given the characteristic findings of HH in PCA described herein, and the fundamental role of static testing for early detection.

The findings also raise questions on the pattern of progression of PCA. Focal forms of neurodegenerative disease are thought to result from a complex interaction between pathology and host-specific local factors (85–87). In the case of AD, evidence supports the hypothesis that, once disease has started, it progresses through connected neurons (88, 89) following a hierarchy of progressive vulnerability (90, 91). It is known that some neurodegenerative diseases tend to be more asymmetric than others [e.g., frontotemporal dementia in comparison with AD (92)] however, as a rule, even in variants characterised by asymmetry, e.g., the primary progressive aphasias, progression involves bilateral changes (93, 94). Likewise, progression toward symmetry has been observed in imaging studies of PCA patients (29). In the present study, the longitudinal correlations between HH and cognitive deficits arising from damage to the same hemisphere may suggest that, at least in initial stages, PCA has progressed faster in one hemisphere. All patients had initially a right parietal syndrome (object perception and/or spatial perception deficits) or biparietal deficits plus an occipital syndrome (HH), with greater impairment in parietal functions related to the hemisphere causing the HH. Progression occurred with worsening of the initial HH and disproportional decline in ipsilateral parietal functions, then development or worsening of contralateral HH, in parallel with the emergence of visual cortical deficits related to bilateral occipital dysfunction. Such a progression suggests that disease may have started in one parietal lobe and progressed toward the ipsilateral occipital and contralateral parietal lobes, or disease may have started in one occipital lobe and progressed to ipsilateral parietal lobe, then to contralateral parietal, before significantly affecting the contralateral occipital lobe. The relative paucity of connections between the two primary visual cortices (95) would explain the tendency to unilateral HH in both cases. However, such speculations assume a similar relationship between neurodegeneration mediated cognitive deficits and neurodegeneration mediated visual field loss, which cannot be taken for granted.

Although they can be compared on a scale of severity, these deficits are very different in nature. Complex cognitive deficits may result from failure in several other individual processes that are required for a given function (96, 97). This may be why imaging correlates of cognitive deficits often involve relatively large brain regions. In contrast, visual fields are a highly quantifiable function, with a point-by-point anatomic correlation. This means the extent of neural damage leading to cognitive deficits may differ from that required for perimetric deficits to appear. From our study on VF progression, we know that HH to static stimulation represents a relative early stage. The occurrence of HH in association with the most common presentations of PCA, i.e., a biparietal syndrome without other occipital deficits, could reflect the higher sensitivity of the deficits measured with HVF – as compared to those reflecting higher-order functions.

### Implications for diagnosis, monitoring and management of patients with PCA/AD

Our principle conclusion is that the occurrence of visual field defects in PCA is an important observation that has profound implications for differential diagnosis and also for our understanding of the underlying pathological process. This applies not only to the identification of PCA as a subtype of AD but also to the phenotypic classification within the PCA diagnosis. Further studies may reveal relevance more broadly in other neurodegenerative disorders associated with visual symptomatology. We would also suggest that the demonstration of stato-kinetic dissociation may prove to be of diagnostic value and also challenges our understanding of the underlying pathology.

Identifying visual loss by quantitative perimetric techniques using static stimuli should be seen as an essential component in the assessment. In individual patients the findings may guide practical and rehabilitation strategies as well as providing an inexpensive, non-invasive and quantitative means of monitoring progression and assessing treatment response. The time it has taken to fully appreciate this aspect of PCA reflects the lack of sensitivity of the neurologists’ classical “hand waving” confrontation perimetry technique. For ophthalmologists and optometrists the finding of such a homonymous hemianopic visual field defect on routine screening in a patient with vague visual symptoms, or even none at all, should lead to an appropriate referral for investigation.

## Limitations of the study

This study has several limitations. The relatively small sample and the great heterogeneity of the patients were expected since PCA is both rare and varied. The longitudinal nature of the study partially compensates for this but it is also the case that the severity of visual field loss in some patients limited the duration of follow-up to one year. Weaker associations may have been missed as a result– e.g. the lack of association between left HH and right hemisphere dysfunction may reflect this rather than genuine independence. Likewise, the association between right HH and left parietal deficits may have been overestimated.

Other limitations are methodological. We did not systematically search for vertical neglect. Vertical neglect has rarely been observed in neurodegenerative disease (60, 98), including PCA (60). However, on cancellation tasks no attention bias in the vertical plane was observed. Dynamic (99) and representational (100) aspects of neglect were also not explored, although it is unlikely that they would affect the perimetric findings. A further issue is that the global scoring system (created to represent the severity of impairment on cognitive functions) consisted of a summary of the results in individual tests that were all validated but was not, itself, validated. Nonetheless the assessment revealed an acceptable degree of consistency. Finally, this study has focused on patients fulfilling criteria for “PCA-pure,” which overwhelmingly represents a variant of Alzheimer’s disease; the findings may not be applicable to PCA due to atypical pathologies, such as LBD or CBD.

## Data Availability

The original contributions presented in the study are included in the article/Supplementary material, further inquiries can be directed to the corresponding author.
